# Exposure to* p*,*p*′-DDE Induces Morphological Changes and Activation of the PKC*α*-p38-C/EBP*β* Pathway in Human Promyelocytic HL-60 Cells

**DOI:** 10.1155/2016/1375606

**Published:** 2016-10-19

**Authors:** Nallely A. Torres-Avilés, Damaris Albores-García, Ana L. Luna, Monica Moreno-Galván, Mariana Salgado-Bustamante, Diana Patricia Portales-Pérez, Emma S. Calderón-Aranda

**Affiliations:** ^1^Departamento de Toxicología, Centro de Investigación y Estudios Avanzados, IPN, Ciudad de México, Mexico; ^2^PIBIOM, ENMH, Instituto Politécnico Nacional, Ciudad de México, Mexico; ^3^Hospital Infantil de México Federico Gómez, SSA, Ciudad de México, Mexico; ^4^Departamento de Bioquímica, Facultad de Medicina, UASLP, San Luis Potosí, SLP, Mexico; ^5^Laboratorio de Inmunología Celular y Molecular, Facultad de Ciencia Químicas, UASLP, San Luis Potosí, SLP, Mexico

## Abstract

Dichlorodiphenyldichloroethylene (*p*,*p*′-DDE), the most persistent metabolite of dichlorodiphenyltrichloroethane (DDT), is still present in the human population. Both are present in the bone marrow of patients with bone marrow disorders, but thus far there are no studies that assess the capability of* p*,*p*′-DDE to affect myeloid cells. The aim of this study was to determine the effect of* p*,*p*′-DDE on promyelocytic cell differentiation and intracellular pathways related to this event.* p*,*p*′-DDE induced morphological changes compatible with promyelocytic differentiation in a concentration-dependent manner. The* p*,*p*′-DDE effect on [Ca^2+^]_i_, C/EBP*β* protein levels, PKC*α* and p38 activation, and the role of oxidative stress or PLA2 was assayed. Exposure to 1.9 *μ*g/mL of* p*,*p*′-DDE increased [Ca^2+^]_i_, PKC*α*, p38, and C/EBP*β* protein levels; the increase of nuclear C/EBP*β* protein was dependent on p38. PKC*α* phosphorylation was dependent on PLA2 and* p*,*p*′-DDE-induced oxidative stress. p38 phosphorylation induced by* p*,*p*′-DDE was dependent on PLA2, PKC activation, and oxidative stress. These effects of* p*,*p*′-DDE at concentrations found in human bone marrow may induce alterations in immature myeloid cells and could affect their cellular homeostasis. In order to establish the risk from exposure to* p*,*p*′-DDE on the development of bone marrow disorders in humans, these effects deserve further study.

## 1. Introduction

Dichlorodiphenyltrichloroethane (DDT) is a persistent organic pollutant that was used until the end of the twentieth century as a pesticide and to control vectors of dengue and malaria worldwide [1]. Because of the high persistence of DDT in the environment and its slow elimination from the body, younger generations in several countries are currently still exposed to DDT and its most persistent metabolite, dichlorodiphenyldichloroethylene (*p*,*p*′-DDE) [2–4].* p*,*p*′-DDE has been found in several human tissues including bone marrow (ranging from 0.46 to 1.6 ppm) [5–7]. Pesticide exposure alters cell differentiation in bone marrow resulting in noncompetent cells or haematological disorders [8–12]. DDT and* p*,*p*′-DDE exposure are associated with a risk of developing bone marrow disorders such as aplastic anaemia, acute myeloid leukaemia (AML), and non-Hodgkin lymphoma [13–19]. Epidemiological data has associated home pesticide exposure during a child's early years with acute lymphoblastic leukaemia (ALL) and exposure before birth with AML [20]. Although epidemiological studies demonstrate the adverse effects of DDT on haematological or immunological parameters [21–25], currently there are no data available on the association between* p*,*p*′-DDE exposure and haematopoietic cell differentiation that could explain either haematological disorders observed in the population or the molecular mechanism underlying DDT toxicity in these cells.

Myeloid differentiation is a process in which committed myeloid progenitors undergo growth arrest and subsequently terminal differentiation into mature granulocytes or monocytes [26]. During myeloid differentiation, cells suffer a characteristic change in their morphology including nuclear restructuring and changes in the size of myeloid precursors [27, 28]. The differentiation of myeloid precursors is accomplished by signalling cascades which are not well understood. CCAAT/enhancer binding protein *β* (C/EBP*β*) is involved in gene expression and regulates differentiation in haematopoietic cells [29]. C/EBP*β* expression is induced in myeloid progenitors when emergency granulopoiesis is required and during monocyte differentiation [30–32]. Its function is regulated by posttranslational modifications including phosphorylation [33]. The C/EBP*β* levels of the cytoplasmic protein (inactive form) and nuclear protein (active form) are controlled by the mitogen-activated protein kinases (MAPK) pathways including extracellular signal-regulated kinases (ERK1/2) (activated by growth factors and survival factors), c-Jun N-terminal kinases (JNK), and p38 (activated by stress stimuli) [34, 35]. It has been shown that protein kinase C (PKC) plays a critical role in MAPK activation [36–38].

The few* in vitro* experiments that have been performed demonstrate that DDT induces adipogenic and osteogenic differentiation of mesenchymal stem cells [39] and adipocyte differentiation from preadipocyte 3T3-L1 cells and increases the nuclear levels of C/EBP*α*,*β* [40]. DDT negatively affects haematic cells and their immunocompetence, but its effect on differentiation of immune cells and the mechanisms underlying these effects remain to be defined. Previous studies in nonmyeloid cells and monocytes have shown that* p*,*p*′-DDT and/or* p*,*p*′-DDE exert different effects on the signalling molecules that participate in myeloid cell differentiation including intracellular calcium levels [Ca^2+^]_i_, reactive oxygen species (ROS) production, activation of MAPK and PKC family, and phospholipase A2 (PLA2) stimulation [41–44]. However, these effects have not yet been evaluated in myelocytic cells.

Considering the impact that DDT exposure could have on haematopoietic differentiation, the goal of this study was to examine the effects of its more persistent metabolite,* p*,*p*′-DDE, on differentiation in the promyelocytic HL-60 cell line and to elucidate which molecular mechanisms implicated in myeloid cell differentiation are altered by* p*,*p*′-DDE exposure. Specifically, this study evaluated the capability of* p*,*p*′-DDE to induce C/EBP*β* stimulation through MAPK activation as well as the participation of PKC activation and ROS, both of which are involved in myeloid differentiation.

## 2. Materials and Methods

### 2.1. Cell Culture

The HL-60 cell line was purchased from American Type Culture Collection (ATCC, USA). The cell line was maintained in RPMI 1640 medium (Sigma-Aldrich Chem. Co., St. Louis, USA) supplemented with 10% fetal bovine serum, 1% penicillin-streptomycin, and 1% L-glutamine (Gibco-BRL, Grand Island, NY). The culture was incubated at 37°C in an atmosphere containing 95% humidity and 5% CO_2_. For all experiments, cells were resuspended in fresh medium and treated with* p*,*p*′-DDE, MAPK inhibitors, or the antioxidant Trolox (Sigma-Aldrich Chem. Co., St. Louis, USA) as indicated. The* p*,*p*′-DDE stock solution was prepared using 1% ethanol as the vehicle, and 5.0–20 *μ*L of the stock solution was added to the cultures to achieve final concentrations of 0.019, 0.19, or 1.9 *μ*g/mL of* p*,*p*′-DDE.

### 2.2. Cell Treatments

Cells were plated in complete medium at a density of 2 × 10^5^ cells/mL. Cells were given 12 h to stabilize before treatments were added; control cells had no treatment and experimental samples received 0.019, 0.19, or 1.9 *μ*g/mL of* p*,*p*′*-*DDE. MAPK inhibitors (PD98059, SP600125, and SD203508 for ERK1/2, JNK, and p38, resp., Sigma-Aldrich Chem. Co., St. Louis, USA) were added 1 h before treatment with* p*,*p*′*-*DDE. Inhibitors for PLA (cPLA2, Calbiochem, USA) or PKC (Myr PKC, Promega, USA) were added 1 h before treatment with* p*,*p*′*-*DDE. Five micrograms of BAPTA-AM (a Ca^2+^ chelator) was added 30 min before treatment with* p*,*p*′*-*DDE. Trolox (Sigma-Aldrich Chem. Co., St. Louis, USA) was added to reach a final concentration of 50 *μ*M 1 h before treatment with* p*,*p*′*-*DDE.

### 2.3. Cell Viability Assay

The 3-(4,5-dimethyl-2-thiazolyl)-2,5-diphenyl tetrazolium bromide (MTT) (Sigma-Aldrich, St. Louis, MO, USA) assay was used for cell viability assay [45]. After 12 h of stabilization, HL-60 cells were treated with 0.019, 0.19, or 1.9 *μ*g/mL of* p*,*p*′*-*DDE for 24, 48, 72, 96, and 120 h. For 1.9 *μ*g/mL of* p*,*p*′*-*DDE cell viability assay was performed also at 144 and 168 h. Absorbance at 590 nm was measured with a spectrophotometer microplate reader (SpectraMax 250, Molecular Devices, Sunnyvale, CA) and the percentage of viable cells was calculated as the absorbance of each experimental condition relative to the absorbance of the control group. Three independent experiments were performed in triplicate.

### 2.4. Cell Proliferation Assay

Cell proliferation was measured by a [^3^H]-thymidine incorporation assay after 24, 48, 72, 96, and 120 h of exposure to* p*,*p*′*-*DDE. Twelve hours before harvesting, 0.5 *μ*Ci/well of [^3^H]-thymidine (Amersham Life Science) was added, and cells were harvested using an automatic Skatron combi cell harvester (Skatron Inst., Lier, Norway) onto fiberglass filters. The incorporation of [^3^H]-thymidine was determined using a beta-plate scintillation counter (Beckman, LS 6500). Three independent experiments were performed in triplicate.

### 2.5. Cytochemistry

For morphological analyses, HL-60 cells grown under different experimental conditions were harvested and centrifuged onto cytospin slides (Shandon CytoSpin 4, Thermo Fisher Scientific, Waltham, MA). According to the manufacturer's instructions, slides were stained with Wright staining solution for 20 min and then observed under a light microscope. Morphological changes of cells were evaluated as cell size (big or small) and nucleus shape (bean or band). Four randomly selected fields containing at least 100 cells were counted. The percentage of cells with morphological changes was calculated from three independent experiments. Monocyte/macrophage differentiation was assayed for nonspecific esterase (NSE) presence using a nonspecific *α*-naphthyl-acetate staining kit (Sigma-Aldrich Chem. Co., St. Louis, USA) according to the manufacturer's recommended protocol. Positive cells to NSE activity were stained reddish-brown colouration.

### 2.6. Determination of CD14 and CD16 by Flow Cytometry Analysis

The expression of cell surface antigens (CD14 and CD16) was determined after 24, 48, 72, 96, and 120 h of exposure to* p*,*p*′*-*DDE by measuring the binding of fluorescein isothiocyanate-CD16 or phycoerythrin-CD14 conjugated antibodies (Abcam, Hong Kong) by flow cytometry. Cells exposed to* p*,*p*′-DDE concentrations were harvested at a density of 10^6^ cells/mL and then incubated with appropriate dilutions of the antibodies according to the manufacturer's instructions. Excess antibody was removed by washing the cells once with PBS (0.01 M phosphate buffered saline and 0.15 M NaCl). Specific antibody binding was measured regarding total fluorescence of the cell population with a four-colour FACS Calibur (Becton Dickinson). The fluorescence was compared to unstained controls with 10,000 events recorded. All data was analysed using FlowJo v7/8 (Tree Star Inc., Ashland, OR).

### 2.7. Measurement of [Ca^2+^]_i_ by Flow Cytometry

[Ca^2+^]_i_ was measured by fluorescence-activated cell sorting (FACS) using Fluo-3-loaded cells (Fluo-3AM, Molecular Probes, Oregon). HL-60 cells were resuspended to a density of 2.5 × 10^5^/mL in PBS containing 13 *μ*M Fluo-3AM and 8% pluronic acid F-127 (Molecular Probes, Oregon, USA) and were incubated for 25 min at 37°C. For treatments with BAPTA-AM (Molecular Probes, Oregon), cells were incubated for 30 min with the Ca^2+^ chelator before incubation with Fluo-3AM. Extracellular Fluo-3AM was removed by washing the cells twice. Finally, the cells were adjusted to a density of 6.25 × 10^4^/mL and resuspended in Ca^2+^-free buffer to evaluate the role of [Ca^2+^]_i_. Ca^2+^ signal was measured with the flow cytometer for 20 seconds to establish a baseline Ca^2+^ level. Subsequently, cells were exposed to different experimental conditions, and the Ca^2+^ signal was measured for up to 3 min. Ionomycin (0.1 *μ*g/*μ*L) and phorbol myristate acetate (PMA) (10 nM) (Sigma-Aldrich Chem. Co., St. Louis, USA) were used to verify the specificity of the signal. Assessment of [Ca^2+^]_i_ was performed at room temperature by flow cytometry (FACS Calibur, Becton Dickinson) using the CellQuestPro software (BD Bioscience) and analysed with FlowJo v7/8 (Tree Star Inc., Ashland, OR).

### 2.8. Western Blot Analysis

For nuclear and cytosolic extracts, cells were washed and harvested with cold TD buffer (12 mM Tris-Base, 68 mM NaCl, 2.5 mM KCl, and 0.4 mM monobasic sodium phosphate, pH 7.4) and pelleted by centrifugation at 1900 rpm at 4°C for 5 min. The pellet was resuspended in two volumes of cold buffer A (10 mM HEPES, 10 mM KCl, 0.1 mM EGTA, 0.1 mM EDTA, 1 mM DDT, 0.5 mM PMSF, 10 *μ*g/mL leupeptin, 5 *μ*g/mL antipain, 5 *μ*g/mL chymostatin, 5 *μ*g/mL benzamidine, and 1 *μ*g/mL pepstatin (Sigma Chem., Co., St. Louis, MO)). After incubation for 15 min at 4°C, 0.5 volumes of 10% NP-40 were added and the samples were mixed for 1 min and centrifuged at 14,000 rpm for 30 s. Cytosolic extracts corresponding to the soluble fraction were retained, and aliquots were stored at −87°C until use. Pellets containing the nuclei were resuspended in extracting buffer C (20 mM HEPES, 0.4 M NaCl, 1 mM EDTA, 1 mM EGTA, 1 mM DTT, 1 mM PMSF, 10 *μ*g/mL leupeptin, 5 *μ*g/mL antipain, 5 *μ*g/mL chymostatin, 1 *μ*g/mL pepstatin, and 5 *μ*g/mL benzamidine). Samples were mixed for 30 min at 4°C followed by centrifugation at 12,000 rpm for 5 min. Nuclear extracts were retained, and aliquots were stored at −81°C until use. Protein concentrations for total, nuclear, and cytosolic fractions were determined with the Bio-Rad protein assay (Bio-Rad Laboratories, Hercules, CA). From each sample, a constant amount of protein (30 *μ*g of total protein or 20 *μ*g of nuclear or cytosolic protein) was separated by 10% SDS-PAGE at 80 V. Proteins were electrotransferred to PVDF membranes (Millipore Corporation, Bedford, MA, USA) in a Hoefer semidry unit (Amersham Biosciences, Buckinghamshire, UK). Blots were blocked for 1 h with 5% fat-free milk in PBS and incubated overnight at 4°C with specific antibodies in PBS containing 3% fat-free milk and 0.1% Tween 20. Specific antibodies were used against phosphorylated PKC*α* and phosphorylated p38 (Santa Cruz Biotechnology, CA, USA), anti-C/EBP*β* (Abcam, Cambridge, USA), anti-histone 4 (Santa Cruz Biotechnology, Santa Cruz, CA), and anti-*β*-actin (kindly donated by Dr. Manuel Hernández's Laboratory, Cinvestav). *β*-Actin and histone 4 were used as loading controls for cytosolic and nuclear proteins, respectively; these proteins were detected on the same membrane after stripping it. Membranes were incubated with anti-rabbit, anti-goat, or anti-mouse biotinylated antibody (Zymed Laboratories Inc., CA, USA), bands were developed using Luminata™ (Millipore, MA, USA) and subsequently visualised using the Odyssey infrared imaging system (LI-COR Bioscience, Lincoln, USA). At least three independent experiments were performed.

### 2.9. Data Analysis

Data were analysed using a one-way ANOVA followed by Dunnett's* post hoc* test or Bonferroni's multiple comparison tests to evaluate cell viability differences over time. All values of *p* < 0.05 were taken to indicate statistical significance. Data analyses were performed using GraphPad Prism version 5.0 (GraphPad Software, Inc., San Diego, CA).

## 3. Results

### 3.1. Viability and Proliferation of HL-60 Cells Exposed to* p*,*p*′*-*DDE

**Figure 1:**
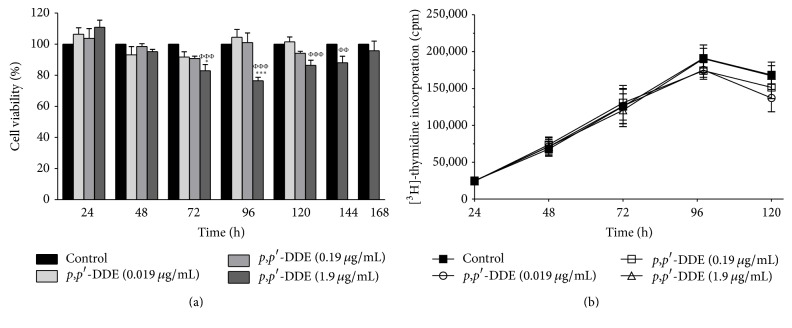
*p*,*p*′*-*DDE exposure does not affect cell viability and proliferation. (a) Percentage of viable cells and (b) proliferation of cells exposed to 0.019, 0.19, and 1.9 *μ*g/mL of* p*,*p*′*-*DDE as evaluated by MTT assay and [^3^H]-thymidine incorporation, respectively. Data from three independent experiments are expressed as the mean ± standard error mean (SEM). ^*∗∗∗*^ *p* < 0.001 indicates differences from the control group; ^ΦΦ^ *p* < 0.01 and ^ΦΦΦ^ *p* < 0.001 indicate differences from 1.9 *μ*g/mL of* p*,*p*′*-*DDE at 24 h. Data were analysed using a one-way ANOVA followed by Dunnett's* post hoc* or Bonferroni's multiple comparison tests. ^*∗*^ *p* < 0.05 indicates differences from the control group.

Viability assays showed a significant decrease in MTT reduction at 72 and 96 h in cells exposed to 1.9 *μ*g/mL of* p*,*p*′*-*DDE compared on control cells, an effect that was no longer present at 120, 144, and 168 h (Figure 1(a)). The cell viability in the presence of 1.9 *μ*g/mL of* p*,*p*′-DDE showed not statistical differences over time, except when compared with 24 h (Figure 1(a)). These data suggest that after the 96 h cells compensate metabolic capability to reduce MTT and reached a steady state. Cell proliferation was not modified by any concentration of* p*,*p*′*-*DDE over 120 h (Figure 1(b)).

### 3.2. *p*,*p*′*-*DDE Exposure Induces Changes in Cell Morphology

**Figure 2:**
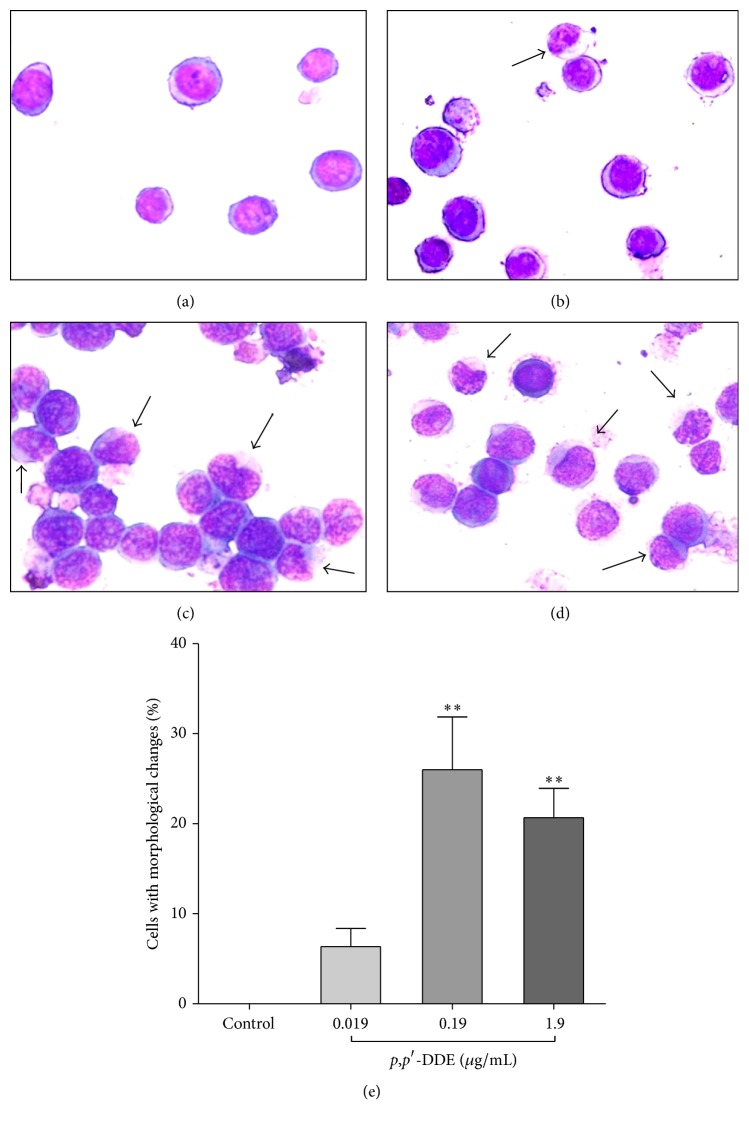
Morphological changes in HL-60 cells induced by* p*,*p*′*-*DDE exposure for 120 h. Representative images of cells with morphological changes (arrows) as evaluated by Wright staining; morphological changes were characterized by a decreased nuclear/cytoplasmic ratio and an irregular nuclear shape. (a) Control cells, (b) cells exposed to 0.019 *μ*g/mL* p*,*p*′*-*DDE, (c) cells exposed to 0.19 *μ*g/mL* p*,*p*′*-*DDE, and (d) cells exposed to 1.9 *μ*g/mL* p*,*p*′*-*DDE. (e) The fraction of cells with morphological changes per 100 live cells after 120 h of treatment. Data from three independent experiments are expressed as the mean ± SEM. ^*∗∗*^ *p* < 0.01 indicates differences from the control group. Data were analysed using one-way ANOVA followed by Dunnett's* post hoc* test.

The morphology of promyelocytic cells was monitored for 120 h to identify any changes induced by 0.019, 0.19, or 1.9 *μ*g/mL of* p*,*p*′*-*DDE. Over the course of 120 h,* p*,*p*′*-*DDE exposure induced morphological changes in a concentration-dependent manner. While control cells presented a circular nucleus (their typical morphology in promyelocytic cells), those treated with 1.9 *μ*g/mL of* p*,*p*′*-*DDE exhibited a kidney-shaped nucleus and had a reduced nuclear/cytoplasmic ratio (Figures 2(a)–2(d)). Morphological changes in cells exposed to 0.19 and 1.9 *μ*g/mL of* p*,*p*′*-*DDE began to appear after only 48 h, and by 120 h the percentage of cells with differentiation changes was three- to fourfold higher than the control (Figure 2(e)).

### 3.3. *p*,*p*′-DDE Exposure Does Not Induce Linage-Specific Markers in Promyelocytic HL-60 Cells

**Figure 3:**
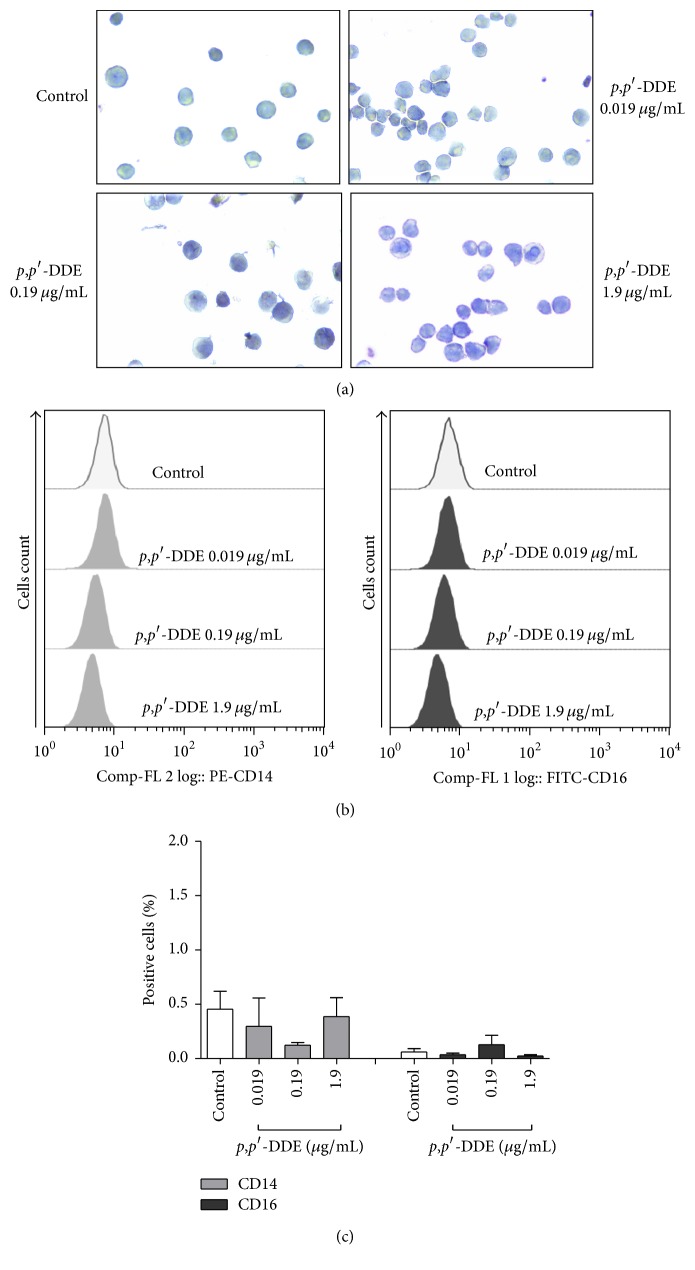
Absence of nonspecific esterase and mature cell surface markers in HL-60 cells exposed to* p*,*p*′*-*DDE. (a) Representative images of nonspecific esterase staining of HL-60 cells exposed to* p*,*p*′-DDE for 120 h. (b) Flow cytometry representative histograms of CD14 and CD16 expression in HL-60 cells exposed for 120 h to* p*,*p*′*-*DDE. (c) The percentages of cells expressing CD14 or CD16 were detected by flow cytometry analyses. The data represent the mean ± SEM of three different experiments. Data were analysed using one-way ANOVA followed by Dunnett's* post hoc* test.

Due to the morphological changes observed in cells treated with* p*,*p*′-DDE, we assessed the presence of NSE as indicative of monocytic differentiation, as well as the expression of linage-specific surface makers. In Figure 3(a) is shown that cells exposed to 0.019, 0.19, or 1.9 *μ*g/mL of* p*,*p*′*-*DDE for five days were negative for NSE. In HL-60 cells treated with* p*,*p*′-DDE (0.019, 0.19, and 1.9 *μ*g/mL), the CD16 and CD14 associated with terminal differentiation of granulocytes and monocytes, respectively, in all experimental conditions were negative to these markers from 24 to 120 h of treatment. In Figures 3(b) and 3(c) are presented results from flow cytometry at 120 h.

### 3.4. *p*,*p*′*-*DDE Exposure Activates p38 Leading to an Increase in C/EBP*β* Protein Levels

**Figure 4:**
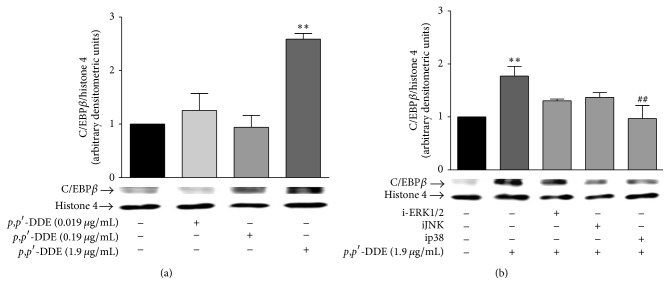
*p*,*p*′*-*DDE exposure increases C/EBP*β* protein levels in the nuclei in a concentration-dependent manner mediated by p38 kinase. (a) C/EBP*β* nuclear protein levels were assessed after 12 h of exposure to 0.019, 0.19, or 1.9 *μ*g/mL of* p*,*p*′*-*DDE. (b) C/EBP*β* nuclear protein levels after 12 h of exposure to 1.9 *μ*g/mL of* p*,*p*′-DDE alone or with iERK (ERK(1/2) inhibitor, PD98058), iJNK (JNK inhibitor SP600125), or ip38 (p38 inhibitor, SD203508). Inhibitors were added 30 min before exposure to* p*,*p*′*-*DDE. Densitometric values were normalized to histone 4 protein levels. Data from three independent experiments are expressed as mean ± SEM. ^*∗∗*^ *p* < 0.01 indicates differences relative to the control and ^##^ *p* < 0.01 indicates differences relative to treatment with 1.9 *μ*g/mL of* p*,*p*′*-*DDE. Data were analysed using one-way ANOVA followed by Dunnett's* post hoc* test.

To examine whether the morphological changes induced by* p*,*p*′*-*DDE exposure are concomitant with activation of the C/EBP*β* transcription factor (associated with myelocytic differentiation), nuclear protein levels were evaluated after 12 h of* p*,*p*′*-*DDE (0.019, 0.19, and 1.9 *μ*g/mL) exposure. Nuclear C/EBP*β* levels increased only upon exposure to 1.9 *μ*g/mL* p*,*p*′*-*DDE (Figure 4(a)).

It is known that C/EBP*β* activation is regulated by MAP kinases such as ERK, JNK, and p38 [46]. Therefore, we assessed the role of these kinases in C/EBP*β* activation induced by exposure to 1.9 *μ*g/mL* p*,*p*′*-*DDE using the MAPK inhibitors iERK, iJNK, and ip38 (for ERK, JNK, and p38, resp.). Only p38 inhibition significantly reduced the C/EBP*β* nuclear protein activation induced by* p*,*p*′*-*DDE exposure (Figure 4(b)).

### 3.5. *p*,*p*′*-*DDE Exposure Increases [Ca^2+^]_i_ in HL-60 Cells

**Figure 5:**
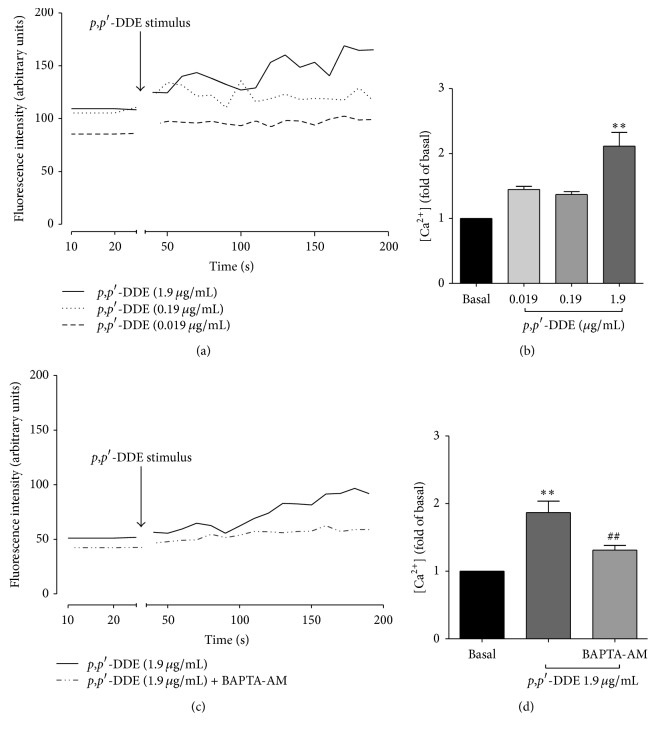
The* p*,*p*′*-*DDE exposure increases [Ca^2+^]_i_. HL-60 cells loaded with Fluo-3-AM in the absence of extracellular Ca^2+^ were used to determine [Ca^2+^]_i_ by flow cytometry. (a) The data shown correspond to a representative experiment measuring [Ca^2+^]_i_ fluorescence where the baseline was registered for 20 s, followed by the addition of* p*,*p*′*-*DDE (as indicated by the arrow) and subsequent monitoring for 200 s. (b) Bars correspond to the maximum increase in [Ca^2+^]_i_ after addition of* p*,*p*′*-*DDE. (c) Fluorescence collection corresponding to [Ca^2+^]_i_ in cells exposed to 1.9 *μ*g/mL of* p*,*p*′*-*DDE with or without BAPTA-AM in the absence of extracellular [Ca^2+^]; results are from a representative experiment. (d) Bars correspond to the maximum increase in [Ca^2+^]_i_. Data from three independent experiments are expressed as the mean ± SEM. ^*∗∗*^ *p* < 0.01 indicates differences relative to baseline levels. ^##^ *p* < 0.01 indicates differences relative to the sample exposed to 1.9 *μ*g/mL of* p*,*p*′*-*DDE. Data were analysed using a one-way ANOVA followed by Dunnett's* post hoc* test.

Since signalling pathways related to p38 phosphorylation and C/EBP*β* activation involve an increase of [Ca^2+^]_i_, the effect of* p*,*p*′*-*DDE (0.019–1.9 *μ*g/mL) on [Ca^2+^]_i_ was evaluated. At 1.9 *μ*g/mL of* p*,*p*′*-*DDE and in absence of extracellular Ca^2+^, [Ca^2+^]_i_ increased twofold over baseline (Figures 5(a) and 5(b)). To verify whether the increase in [Ca^2+^]_i_ was caused by* p*,*p*′*-*DDE, the Ca^2+^ chelator BAPTA-AM was used. As shown in Figures 5(c) and 5(d), BAPTA-AM completely blocked the* p*,*p*′*-*DDE-induced increase in [Ca^2+^]_i_.

### 3.6. Effect of* p*,*p*′*-*DDE on PKC*α*, p38, ROS, and Phospholipase A

**Figure 6:**
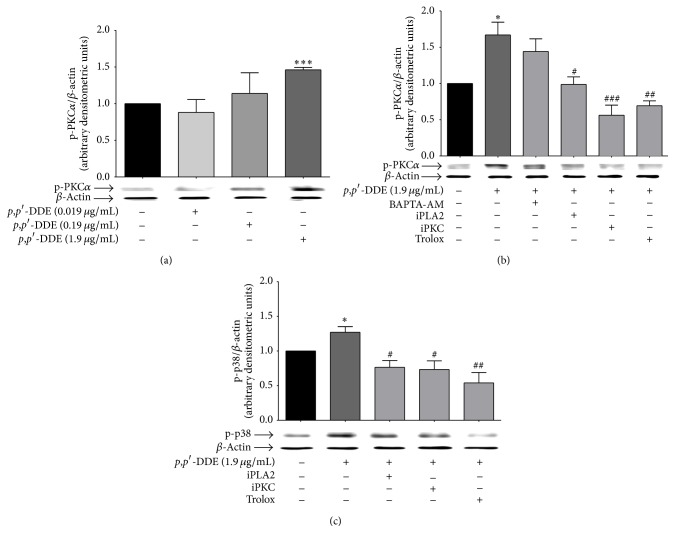
*p*,*p*′*-*DDE induces PKC*α* and p38 activation through PLA2 and oxidative stress. (a) Western blot and densitometric analysis of PKC*α* phosphorylation after 1 h of exposure to 0.019, 0.19, or 1.9 *μ*g/mL of* p*,*p*′*-*DDE. (b) PKC*α* and (c) p38 phosphorylation in HL-60 cells exposed for 1 h to 1.9 *μ*g/mL of* p*,*p*′*-*DDE in cultures pretreated with the compounds indicated below each bar: BAPTA-AM (intracellular calcium chelator), PLA2 inhibitor (iPLA2), PKC inhibitor (iPKC), or Trolox (antioxidant). Data is presented as the mean ± SEM from three independent experiments. ^*∗*^ *p* < 0.05 and ^*∗∗∗*^ *p* < 0.001 indicate differences relative to basal levels; ^#^ *p* < 0.05, ^##^ *p* < 0.01, and ^###^ *p* < 0.001 indicate differences relative to cells exposed to 1.9 *μ*g/mL of* p*,*p*′*-*DDE. Data were analysed using a one-way ANOVA followed by Dunnett's* post hoc* test.

Because the activation of PKC*α* has been shown to be involved in MAPK's signalling to induce haematopoietic differentiation, the effect of* p*,*p*′*-*DDE on PKC*α* activation was evaluated. Only exposure to 1.9 *μ*g/mL of* p*,*p*′*-*DDE induced a significant increase in PKC*α* activation (Figure 6(a)). To determine whether the* p*,*p*′*-*DDE-induced PKC*α* activation is related to the increase in [Ca^2+^]_i_, PKC*α* activation was evaluated in cells treated with BAPTA-AM prior to treatment with 1.9 *μ*g/mL of* p*,*p*′*-*DDE. BAPTA-AM blocked the* p*,*p*′*-*DDE-induced increase in [Ca^2+^]_i_, but* p*,*p*′*-*DDE-induced PKC*α* phosphorylation was not modified by the chelator (Figure 6(b)). To evaluate whether* p*,*p*′*-*DDE is capable of inducing PKC*α* activation through PLA2 activation or oxidative misbalance, the antioxidant Trolox and a PLA2 inhibitor were used. Both Trolox and the PLA2 inhibitor significantly decreased the PKC*α* phosphorylation induced by 1.9 *μ*g/mL of* p*,*p*′*-*DDE (Figure 6(b)). The roles of PKC*α*, ROS, and PLA2 in the p38 phosphorylation were evaluated in cells treated with 1.9 *μ*g/mL of* p*,*p*′*-*DDE for 1 h.* p*,*p*′*-*DDE exposure significantly increased p38 phosphorylation when compared to control cells. Inhibitors of PKC*α*, PLA2, and Trolox significantly decreased the p38 phosphorylation induced by 1.9 *μ*g/mL of* p*,*p*′*-*DDE (Figure 6(c)).

## 4. Discussion

The presence of* p*,*p*′*-*DDE in bone marrow [5–7, 47] has been correlated with alterations in cell differentiation resulting in haematological disorders [16, 18, 19]. In the present study, the cell viability results suggest that concentrations of* p*,*p*′-DDE used were not cytotoxic. At the higher concentration (1.9 *μ*g/mL* p*,*p*′-DDE), a transient decrease in cells seems to occur at 96 h; however cultures compensated the effect and reached the steady state after this time. In fact, because results did not show an effect of 1.9 *μ*g/mL* p*,*p*′DDE on proliferation at 96 h, we considered that the effect observed in MTT assays at this time is a consequence of a temporal decrease of metabolic capability of cells to reduce of MTT instead of cell death. Morphological changes in promyelocytic HL-60 cells were observed after a five-day exposure to 0.19 and 1.9 *μ*g/mL of* p*,*p*′*-*DDE, suggesting that noncytotoxic concentrations of* p*,*p*′*-*DDE induce incomplete differentiation. These observations agree with the ability of* p*,*p*′*-*DDT (at 20 *μ*M) to induce differentiation in adipocytes 3T3-L1 and preadipocytes 3T-F442A [40]. They are also consistent with a recent study in human mesenchymal stem cells (MSCs) from bone marrow, which showed that* p*,*p*′*-*DDT exposure enhanced osteogenic and adipogenic differentiation [39]. Our work suggests that* p*,*p*′*-*DDE exposure at the concentrations found in bone marrow of humans can induce intracellular molecular pathways (ROS, PKC*α*, p38, and C/EBP*β*) that are involved in the myeloid differentiation and, consequently, induce morphological differentiation of this human myeloid cell line. The* p*,*p*′*-*DDE-induced morphological changes of HL-60 cells are compatible with early differentiation events of commitment in promyelocytic HL-60 where cells with a regular and prominent nucleus are transformed into smaller cells with an irregularly shaped nucleus [48]. In our study, promyelocytic cells were transformed by* p*,*p*′*-*DDE exposure into cells with a dentated nucleus, which is a change that takes place when promyelocytic HL-60 cells transform into metamyelocyte precursors without becoming fully mature cells. This change occurred without alterations in cell proliferation, suggesting that* p*,*p*′*-*DDE exposure induces only partial differentiation; neither the presence of lineage-specific surface markers of monocytes or granulocytes (CD14 and CD16) nor the presence of nonspecific esterases was detected. Previously, it has been shown that another organochlorine pesticide, heptachlor, induced monocyte-macrophage differentiation in a myeloid leukaemia cell line [49]. However, the underlying molecular pathways or mechanisms involved have not been defined. Also, it is noteworthy that exposure to heptachlor is associated with an increased risk of developing leukaemia and non-Hodgkin lymphoma [50, 51]. Similarly, there is data suggesting that DDT and* p*,*p*′*-*DDE exposure are a risk factor for bone marrow disorders such as aplastic anaemia, AML, and non-Hodgkin lymphoma [13–19].

To evaluate the mechanisms by which* p*,*p*′*-*DDE induced differentiation in promyelocytic HL-60 cells, the effect of this compound on several molecular targets was studied. We evaluated the effect of* p*,*p*′*-*DDE on intracellular molecules that are involved in activation of C/EBP*β*, a transcription factor activated during myeloid differentiation [30–32]. Our results demonstrate that* p*,*p*′*-*DDE induced an increase in nuclear levels of C/EBP*β*, an effect that was dependent on p38 activation and is consistent with data previously reported [52]. The effect of* p*,*p*′*-*DDE on C/EBP*β* was similar to what occurs in myelopoiesis triggered by external stimuli (such as in response to an infection) and some kinds of proinflammatory diseases such as rheumatoid arthritis [30, 53]. ERK and JNK may also mediate the transcription activity of C/EBP*β* in different cell types [35, 54], but we found that the* p*,*p*′*-*DDE-induced increase in C/EBP*β* nuclear levels was only dependent on p38. In HL-60 cell differentiation induced by PMA, the activation of PKC results in p38 activation [55, 56]. Our data demonstrate that p38 phosphorylation induced by* p*,*p*′*-*DDE is dependent on PKC*α* activation. Because it is known that the p38 pathway is activated by oxidative stress stimuli [34, 57] and in HL-60 cells the p38 activity is induced by ROS [58, 59], the relationship between the rise of p38 phosphorylation with ROS production was evaluated using an antioxidant. We found that p38 phosphorylation induced by* p*,*p*′*-*DDE was ROS-dependent.

To determine the pathway through which* p*,*p*′*-*DDE induces PKC*α* activation, we evaluated the impact of* p*,*p*′*-*DDE on [Ca^2+^]_i_. Our results demonstrate that exposure to 1.9 *μ*g/mL of* p*,*p*′*-*DDE raises the [Ca^2+^]_i_ level, which is known to occur in other cell types as well [42, 60–62]. [Ca^2+^]_i_ chelation partially, but not significantly, inhibited* p*,*p*′*-*DDE-induced PKC*α* activation. Instead, our data revealed that the activation of PKC*α* induced by* p*,*p*′*-*DDE is ROS-dependent. Previous studies have reported that* p*,*p*′*-*DDE is capable of inducing ROS in blood mononuclear cells at concentrations of 60 and 80 *μ*g/mL [63]. Because ROS has been associated with activation of some pathways upstream of PKC*α*, like PLA2 [64, 65], the role of PLA2 was explored. Our results showed that, in addition to ROS, PKC*α* activation induced by* p*,*p*′*-*DDE was also PLA2-dependent.

It has recently been reported that other organochlorine pesticides, such as Aldrin and* trans*-nonachlor, induced PLA2 and PKC*α* activity in HL-60 promyelocytes, human THP-1 monocytes, and murine J774A.1 macrophages [44]. It is important to note that PLA2 plays a role in p38 activation induced by* p*,*p*′*-*DDE since inhibition of PLA2 prevents PKC*α* activation which interferes with the p38 phosphorylation induced by* p*,*p*′*-*DDE. This situation is analogous to that described in monocytes [66].

**Figure 7:**
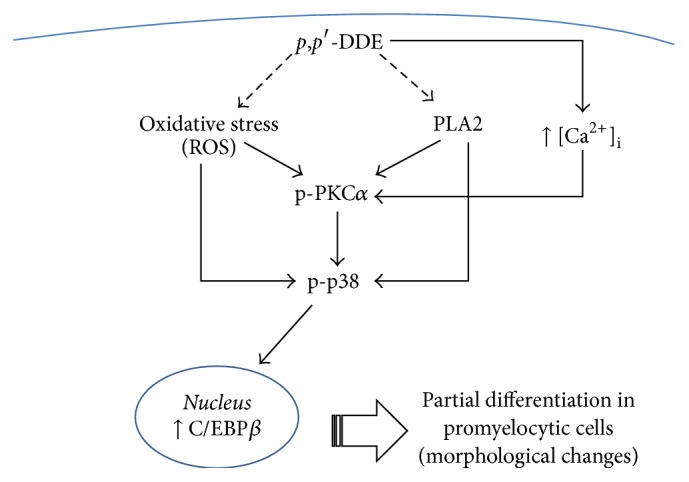
Schematic representation of the* p*,*p*′*-*DDE-induced effects in promyelocytic HL-60 cells.* p*,*p*′*-*DDE induces PKC*α* and p38 activation as well as augmentation of the levels of C/EBP*β* in nuclei. Calcium ions, oxidative stress, and PLA2 participate in the activation of the PKC*α*-p38 signalling pathway. A biologically plausible relationship between* p*,*p*′*-*DDE and its effects on PKC*α*, p38, and C/EBP*β* and morphological changes in promyelocytic HL-60 cells are indicated.

In summary, this study supports the fact that* p*,*p*′*-*DDE exposure in the concentration range found in human bone marrow [5–7] is capable of inducing partial differentiation in HL-60 cells. Our results are consistent with the possibility that PLA2 and ROS trigger signalling pathways involved in PKC*α* activation, whereas ROS and PKC*α* induction are the most important stimuli for p38 activation and consequently increase C/EBP*β* protein levels in the nucleus of HL-60 cells exposed to* p*,*p*′*-*DDE (Figure 7). These findings offer a putative molecular mechanism to partially explain the potential consequences of having* p*,*p*′*-*DDE in human bone marrow and suggest that exposure to this pesticide could potentially lead to the development of bone marrow disorders in human. Further studies are necessary to conclusively support this hypothesis.
